# Impacts of IOD, ENSO and ENSO Modoki on the Australian Winter Wheat Yields in Recent Decades

**DOI:** 10.1038/srep17252

**Published:** 2015-11-30

**Authors:** Chaoxia Yuan, Toshio Yamagata

**Affiliations:** 1 Key Laboratory of Meteorological Disaster of Ministry of Education, Collaborative Innovation Center on Forecast and Evaluation of Meteorological Disasters, Nanjing University of Information Science & Technology, Nanjing 210044, China; 2 Application Laboratory, JAMSTEC, Yokohama 236-0001, Japan

## Abstract

Impacts of the Indian Ocean Dipole (IOD), two different types of El Niño/Southern Oscillation (ENSO): canonical ENSO and ENSO Modoki, on the year-to-year winter wheat yield variations in Australia have been investigated. It is found that IOD plays a dominant role in the recent three decades; the wheat yield is reduced (increased) by −28.4% (12.8%) in the positive (negative) IOD years. Although the canonical ENSO appears to be responsible for the wheat yield variations, its influences are largely counted by IOD owing to their frequent co-occurrence. In contrast, the ENSO Modoki may have its distinct impacts on the wheat yield variations, but they are much smaller compared to those of IOD. Both the observed April-May and the predicted September-November IOD indices by the SINTEX-F ocean-atmosphere coupled model initialized on April 1st just before the sowing season explain ~15% of the observed year-to-year wheat yield variances. The present study may lead to a possible scheme for predicting wheat yield variations in Australia in advance by use of simple climate mode indices.

Australia is one of the major wheat exporting countries in the world and accounts for 10–15% of the global wheat trades. The major exporting destinations include Indonesia, Korea, Sudan, China and Philippines. Also, one fifth of Japan’s wheat import is dependent on Australia, especially the granary of Western Australia. Despite the consistent oversea demand, the winter wheat yield undergoes considerable year-to-year fluctuations. These fluctuations are mainly driven by climate factors, especially precipitation and surface temperature^1^, in the wheat growing seasons spanning from May to November. Hence, understanding and estimating the climate-related wheat yield variations is highly important for the world food management and security.

Tropical climate modes such as El Niño/Southern Oscillation (ENSO) and Indian Ocean Dipole (IOD) catalogued in 1999^2^ are known to be responsible for the climate variations in Australia^3^,^4^. El Niño is often related to a large-scale drought condition, whereas La Niña introduces more precipitations especially in eastern Australia. Hence, the ENSO indices have often been applied to estimate the year-to-year wheat yield variations^5^,^6^,^7^. In general, the occurrence of El Niño (La Niña) reduces (increase) the wheat yield. Also, the April-May trend of ENSO indices show some prediction skills for the wheat yield in the subsequent growing seasons^6^. However, the ENSO influences on the Australian climates are not stable and can be modified by decadal/inter-decadal climate modes such as Pacific Decadal Oscillation^8^. In the recent decades, it is found that IOD may play a more important role than ENSO; the severe drought since 1995 referred to as “Big Dry” has been attributed to less occurrences of the negative IOD rather than La Niña or more occurrence of the positive IOD^9^,^10^. Meanwhile, probably due to the global warming^11^, the tropical Pacific favors the occurrences of ENSO Modoki, which show different teleconnection patterns to Australia compared to the canonical ENSO^12^,^13^,^14^. Hence, it is important to examine impacts of the tropical climate modes on the Australian wheat yields by addressing the new climate modes. This will provide useful information to farmers and decision makers for the crop management.

In this study, we compare the impacts of IOD, canonical ENSO and ENSO Modoki on the Australian year-to-year winter wheat yield variations at the national level in the recent three decades by using linear correlation, regression and composite analyses. Also, we examine the seasonal predictions of the wheat yield variations based on the observed tropical climate mode indices or the predicted ones by the SINTEX ocean-atmosphere coupled model right before the sowing date.

## Results

**Figure 1:**
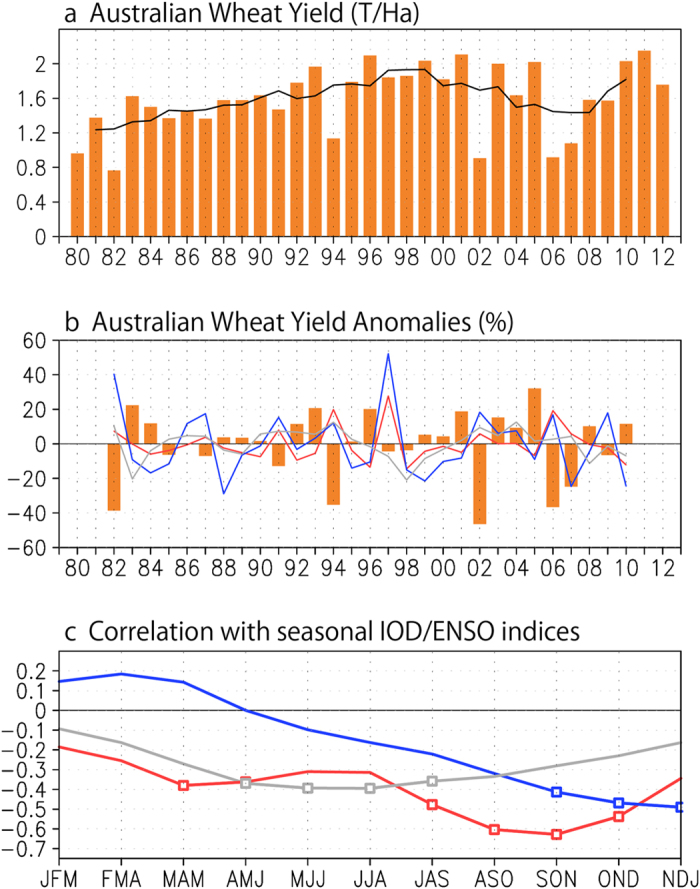
Time series of (**a**) the Australian winter wheat yields, (**b**) their year-to-year anomalous percentages (%) and (**c**) correlation coefficients with the three-month-running mean indices of IOD (DMI, red line), canonical ENSO (Niño3, blue line) and ENSO Modoki (EMI, gray line). The correlation coefficients significant at the 95% confidence level are marked by the open squares in c. The September-November DMI (red, ˚C), November-January Niño3 (blue, ˚C) and June-August EMI (gray, ˚C) are superimposed in b after multiplied by 15. Years in the X axis in a-b denote the years when the wheat is sowed.

As shown in Fig. 1, the Australian wheat yield shows an apparent linear trend in the recent three decades probably owing to improvement in the agricultural technology. Besides, it undergoes considerable year-to-year variations. Here, the year-to-year variations are referred to the anomalous percentage deviated from the five-year running mean^7^. We have calculated the linear correlation coefficients with the seasonal IOD indices (DMI), canonical El Niño indices (Niño3) and ENSO Modoki indices (EMI) before and during the growing seasons. Results show that the year-to-year wheat yield variations have the highest coefficient of −0.64 with DMI in September-November (SON) at the 99% confidence level by the two-tailed *t* test. With Niño3, the highest coefficient is −0.49 in November-January (NDJ) at the same confidence level. With EMI, it is −0.39 in June-August (JJA), significant at the 95% confidence level. We note that the tropical climate modes may impact the wheat continuously throughout the whole growing seasons. The seasons of SON, NDJ and JJA are selected here not because IOD and ENSO have the strongest influences in these specific seasons, but because the indices in these specific seasons may represent the integrated impacts of the tropical climate modes on the wheat yields. It is apparent that IOD has the highest correlation coefficient with the year-to-year wheat yield variations, which implies the most important role of IOD and seems to be consistent with the recent remarkable impacts of IOD on the Australian precipitations^9^,^10^. Although the present study has focused on the wheat yield variations at the national level, we note that IOD also shows higher correlation coefficients than ENSO at the provincial levels (Supplementary Figs 1–2).

**Figure 2:**
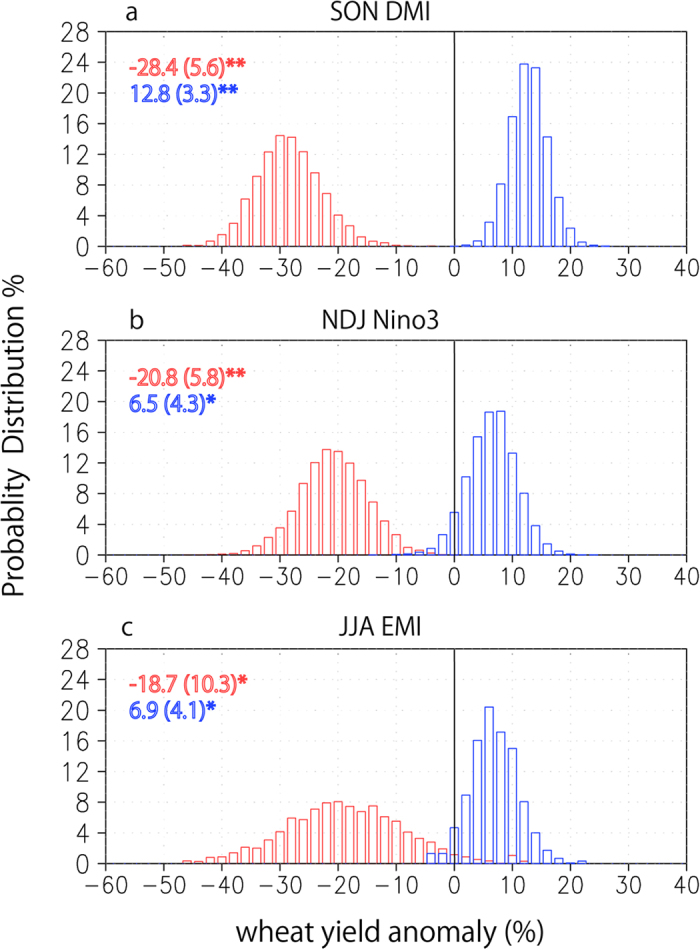
Probability distribution (%) of the mean winter wheat yield anomaly (%) in Australia in positive (red) and negative (blue) years of (**a**) IOD, (**b**) canonical ENSO and (**c**) ENSO Modoki after 10,000 bootstrap samplings. The positive and negative years are selected based on the 0.7 standard deviation of September-November DMI, November-January Niño3 and June-August EMI, respectively. Red and blue numbers with (without) parenthesis in each panel denote the average (standard deviation) of mean yield anomalies in the positive and negative event years. Double (single) asterisks denote the confidence level at 99% (90%).

The composite wheat yield anomalies in the positive/negative IOD and ENSO years are also investigated. The event years of IOD/canonical ENSO/ENSO Modoki are selected when the time series of SON DMI/NDJ Niño3/JJA EMI cross 0.7 standard deviations of the monthly indices, corresponding to 0.3/0.7/0.4 °C, respectively. A conventional technique of bootstrap sampling is applied for 10,000 times and the probability distribution of the averaged wheat yield anomalies in the event years are obtained as shown in Fig. 2. In the positive (negative) IOD years, the averaged wheat yield anomaly is −28.4% (12.8%). In the canonical El Niño (La Niña) years, the anomaly is −20.8% (6.5%). Similar results are obtained in the El Niño (La Niña) Modoki years with the averaged anomaly of −18.7% (6.9%). The smaller anomalous values in the two kinds of ENSO years compared to the IOD years are consistent with the correlation analysis discussed earlier and may be due to their different impacts on the wheat-growing season precipitations. As shown in Supplementary Fig. 3, in the two kinds of El Niño years, the precipitation anomalies are confined to a smaller portion of the wheat belt from May to September compared to those in the positive IOD years. In the canonical La Niña and La Niña Modoki years, the precipitation increases mostly in eastern Australia, and thus significant increase in the wheat yields is only found in Queensland, New South Wales and Victoria (Supplementary Figs 4). In contrast, during the negative IOD years, the region with the increased precipitations is much broader in July-September, extending from northwestern to southeastern Australia. This suggests a significant increase of the wheat yield in most of the major wheat producing provinces except Western Australia.

Owing to the frequent co-occurrence, the IOD and ENSO impacts on the Australian wheat yields seem to have been mixed up in the past. However, we now know climate modes occur independently in some years. Therefore it is interesting to conduct the multi-linear regression analysis using SON DMI, NDJ Niño3 and JJA EMI as predictors and the year-to-year wheat yield variations as predictand. This gives


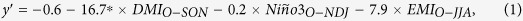


where the asterisk denotes the coefficient statistically significant at the 95% confidence level and the subscript *o* denotes the observed indices. The regression equation (1) explains ~44.3% of the total year-to-year wheat yield variances. It is apparent that IOD plays the dominant role in the last three decades; using SON DMI alone as the predictor can explain ~39.4% of the total year-to-year variances. In contrast, the impacts of ENSO on the wheat yield variations are insignificant in the equation, suggesting that they are mostly counted by the co-occurring IOD. Actually, when the common components between SON DMI and NDJ Niño3 are linearly removed, the correlation coefficient between the year-to-year wheat yield variations and the residual of NDJ Niño3 is only −0.08, while that with the residual of SON DMI remains −0.41. Similarly, when the common components between SON DMI and JJA EMI are removed, the correlation coefficient between the wheat yield variations and the residual of JJA EMI becomes −0.18, while that with the residual of SON DMI remains −0.56. Hence, compared to the canonical ENSO, the ENSO Modoki may retain some independent impacts on the Australian wheat yields, but those are much smaller than the IOD impacts.

**Figure 3:**
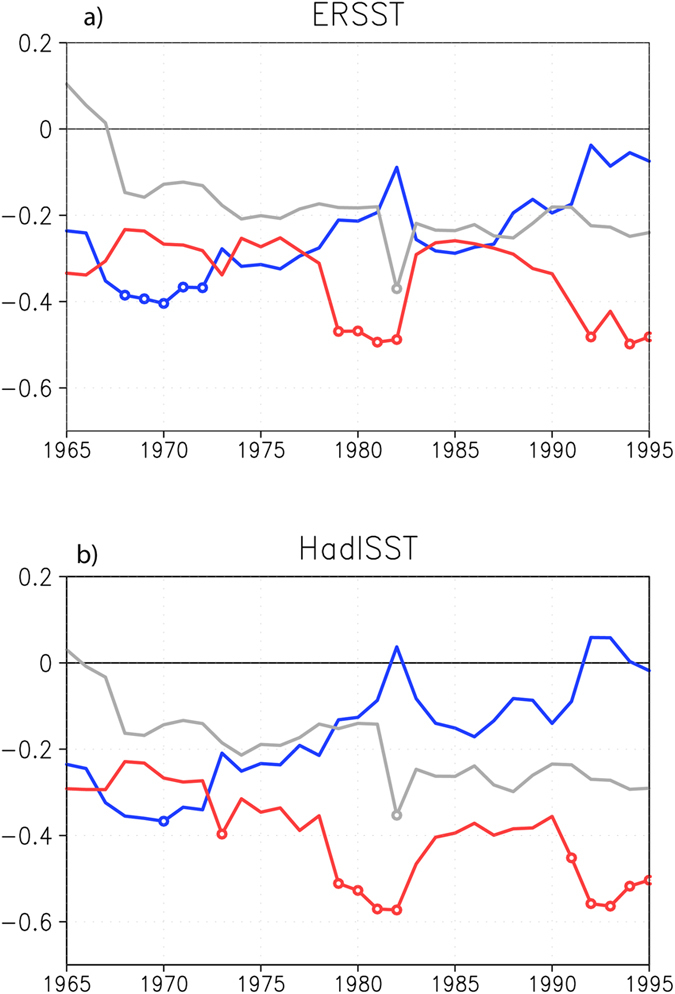
31-year sliding multi-linear regression coefficients of the Australian winter wheat yield variations with SON DMI (red), NDJ Niño3 (blue), and JJA EMI (gray) based on the observed wheat yield and (**a**) ERSST and (**b**) HadISST. The coefficients significant at the 95% confidence level are marked by open circles. The year in X axis denotes the central year of the 31-year sliding window.

**Figure 4:**
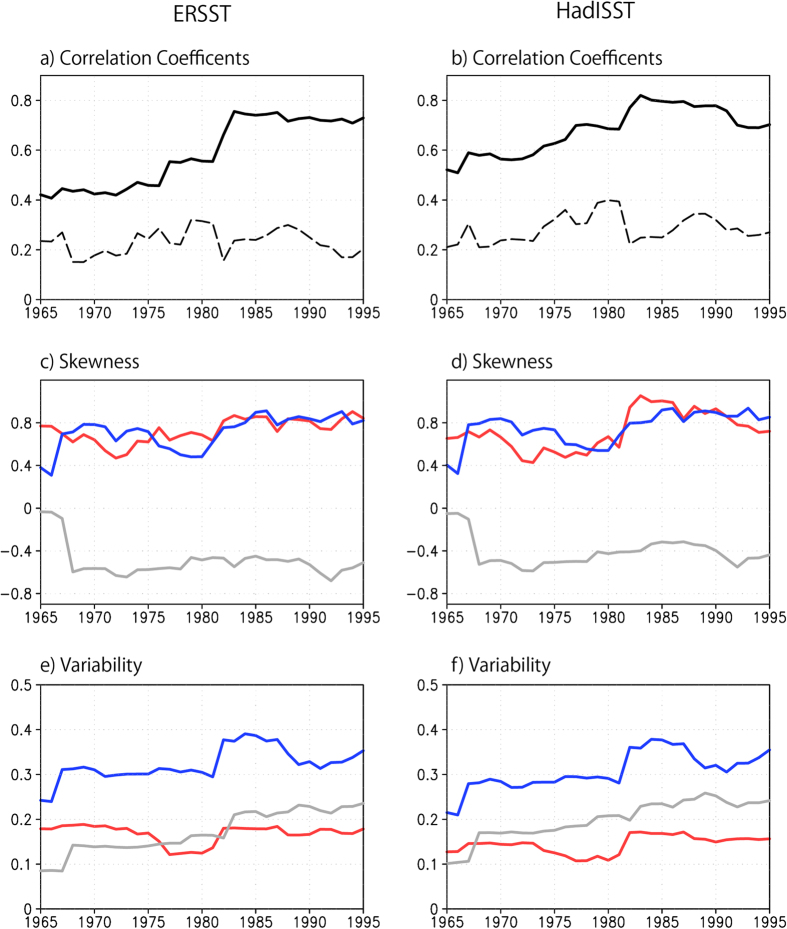
(**a,b**) Correlation coefficients of SON DMI with NDJ Niño3 (solid line) and JJA EMI (dotted line), (**c,d**) skewness and (**e,f**) variability of SON DMI (red), NDJ Niño3 (blue) and JJA EMI (gray) based on the 31-year sliding windows with (**a,c,e**) ERSST and (**b,d,f**) HadISST. The skewness of JJA EMI in (**c,d**) has been divided by 2 and the variability of NDJ Niño3 in (**e,f**) has been divided by 4 for a better comparison. The year in X axis denotes the central year of the 31-year sliding window.

As shown in Fig. 3, the multi-linear regression coefficients based on the 31-year sliding windows since 1950 further confirm the stronger influences of IOD on the Australian wheat yield in the recent decades. The coefficient related to IOD has a clear increasing trend. In contrast, the coefficient related to the canonical ENSO is approaching zero with time. This does not mean that the potential impacts of the canonical ENSO on the Australian wheat are declining. Rather, they are counted by the frequent co-occurring IOD impacts. As shown in Fig. 4a,b, the correlation coefficient between the DJF Niño3 and SON DMI has been increasing since 1950 and reaches ~0.7 in the last three decades. Cai *et al.* have shown that the teleconnection of canonical El Niño to extratropical Australia is via the emanated Rossby waves by the eastern tropical Indian Ocean SST anomalies, which can be counted by the positive IOD^10^. Since the positive skewness of canonical ENSO has been increasing since 1950 (Fig. 4c,d), this may explain why the impacts of canonical ENSO on the Australia wheat yields are more and more counted by IOD. On the other hand, the ENSO Modoki shows a negative skewness. Since there are less co-occurrence between La Niña Modoki and the negative IOD (Supplementary Table 1), the influences of ENSO Modoki on the wheat yields may have some independence from IOD, and they may increase in future with increasing variability (Fig. 4e,f).

In 2007, the canonical La Niña occurred. People expected that the severe drought in Australia since 2006 might ease. However, the drought condition was even worsened especially in southeastern Australia due to less precipitation associated with the co-occurring positive IOD. The remarkable reduction in the actual wheat yields generated a big societal issue^15^,^16^. This is again consistent with the dominant role of IOD in the Australian wheat yields in the recent decades. Since Australia is responsible for a large portion of global wheat trade, skillful predictions of IOD several seasons ahead and its impacts on the Australian wheat yields in advance are of particular importance not only for the domestic wheat growers but also for the food price and security in those major exporting destinations. Although SON DMI may represent the influences of IOD on the wheat yield variations, the April-May (AM) DMI can also give some hints beforehand (Fig. 1b). This is probably because some interannual IOD events develop much earlier in AM and peak in austral winter^17^. The linear equation based on the wheat yields and *observed* AM DMI gives


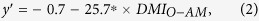


explaining 14.5% of the observed year-to-year wheat yield variances, less than half of that explained by the observed SON DMI.

Another approach to predict the IOD-related wheat yield variations is by using the *predicted* SON DMI. The operational seasonal prediction system based on the SINTEX-F ocean-atmosphere coupled model is one of the best systems predicting IOD^18^,^19^. The hindcast predictions of SON DMI from 1982–2010 with the initialization on April 1^st^ right before the sowing date are applied to estimate the subsequent growing-season wheat yield variations. The linear regression equation based on the *predicted* SON DMI gives


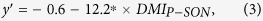


where the subscript *p* denotes the predicted SON DMI. The equation (3) explains ~15.3% of the observed year-to-year wheat yield variances. Even if the predicted JJA EMI and NDJ Niño3 with the same initialization on April 1^st^ are included in the linear equation, the explained total variance is only slightly increased to ~17.7%. This suggests the prediction model simulates the IOD importance successfully to some extent. We must admit that the predicted SON DMI is not superior to the observed AM DMI in predicting the wheat yield variations due to the limited prediction skills of the negative IOD (Supplementary Fig. 5). Nevertheless, the prediction result provides some useful information before the planting date and encourages more efforts on improving the IOD predictions in the future. Actually, there is much space left for improving dynamical seasonal predictions.

## Discussion

In the present study we have illustrated the dominant role of IOD compared to the two types of ENSO in the recent decades. We have clarified respective roles of climate modes originated in the Indo-Pacific Oceans on the year-to-year variations of the Australian winter wheat yields for the first time. Then, we have demonstrated promising skills in predicting the wheat yield variations using the simple linear regression equation based on the observed or predicted IOD indices.

The climate in Australia is influenced not only by the tropical climate modes but also by systems originated in mid and high latitudes^20^. In 2010 when the negative IOD and canonical La Niña co-occurred, the wheat yields in most regions of the wheat belts were either greatly increased or remained normal except Western Australia (Supplementary Figs 2a–l). In fact, the year of 2010 is an outlier in regard to the IOD impacts on Western Australia; if this year is excluded from the statistical analyses, the average anomalies in the wheat yields are significantly positive in the negative IOD years (Supplementary Fig. 6). Also, the correlation coefficient between SON DMI and the Western Australian wheat yield variations becomes −0.38, significant at the 95% confidence level. The exception of 2010 is because southwestern Western Australia, where the major wheat fields are located (Supplementary Fig. 1), experienced the driest condition on record. According to Bureau of Meteorology, Australia, the persistence positive phase of Southern Annual Mode (SAM) from March 2010 till February 2011 is likely to have contributed to the driest conditions. Since the SAM is basically an atmospheric intrinsic mode, the prediction skill of SAM is generally low except for the cases related to the ENSO teleconnection^21^. Hence, improving skills for predicting the impacts of the mid-high latitude climate systems is also important for better estimation of the wheat yield variations in Australia.

We have shown that the SINTEX-F seasonal prediction system has some skills predicting the Australian winter wheat yield variations when initialized on April 1^st^. This is consistent with the work of Iizumi *et al.* 2013^22^ where they have used the predicted local climate factors by the same model to regress the wheat yield variations, and have concluded that the Australian wheat yields can be skillfully predicted at 3–6 months ahead. In this study we have focused on more concrete impacts of climate modes rooted in the Indo-Pacific Oceans. Recent studies reveal that the SST anomalies in the tropical Atlantic may trigger the occurrence of ENSO^23^,^24^. This indicates that the tropical Atlantic may have some indirect influences on the Australian wheat yields at least via the tropical Pacific. Here, we have discussed more direct impacts of the Indo-Pacific climate modes on the Australian wheat yields.

Although the approach presented in this article is still simple, we believe that it may shed new light on the wheat strategy, and possibly benefits a large population in Asia-Oceania regions. It is very important to proceed further in this promising direction. We believe that our science and technology for dynamical climate prediction is close to open a door to an innovative world utilizing societal as well as industrial predictands beyond physical variables.

## Methods

The national and state mean wheat yield data from 1948/49 to 2012/13 is downloaded from the Australian Bureau of Statistics. The year-to-year wheat yield variations 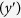 are defined as the anomalous percentage deviated from the five-year running mean, 
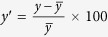
, where *y* is the annual wheat yield and 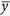 the five-year running mean^7^. The deviation from the five-year running mean rather than the long-term mean is chosen here to stress the anomalies related to the climate variations and to minimize other influential factors such as improvement in cropping technology. However, even if the deviation from the long-term mean is adopted, the conclusions are the same.

Monthly sea surface temperatures (SSTs) during January 1982 to December 2011 from Optimally Interpolated Sea Surface Temperature^25^ (OISST v2) are used for the recent three decades. For the long-term dataset since 1950, the SSTs are adopted from the NOAA ERSST v3^26^ and HadISST^27^. The IOD index (DMI) is defined as difference in SST anomalies between its western (50˚–70˚E, 10˚S-10˚N) and eastern (90˚–110˚E, 10˚S-Eq) poles^2^. The SST anomalies averaged in Niño3 region (90˚–150˚W, 5˚S-5˚N) is used to represent the canonical ENSO. The ENSO Modoki index (EMI) is defined as EMI = [SSTA]_A_– 0.5 x[SSTA]_B_–0.5 x[SSTA]_C_, where A, B and C represent the regions of (165˚E-140˚W, 10˚S-10˚N), (110˚W-70˚W, 15˚S-5˚N) and (125˚E-145˚E, 10˚S-20˚N)^11^. We note that the SST anomalies here are the deviations from the 30-year mean, and DMI/Niño3/EMI indicates the interannual variations. Also, all the three indices have been linearly detrended beforehand. In this study, IOD/canonical ENSO/ENSO Modoki events are selected when the three-month-mean indices cross 0.7 standard deviations (SD), corresponding to 0.3/0.7/0.4 °C, respectively.

The linear relationships between the climate modes and yield anomalies are examined by the linear correlation/regression analyses and the statistical significance is tested by the two-tailed *t* test. The detailed impacts of the climate modes are also investigated by the bootstrap method. The bootstrap sampling of 29 years is repeated for 10,000 times, and at each time, the mean yield anomalies in the positive/negative event years are calculated. The probability distribution of the mean yield anomalies in the positive/negative event years for the 10,000 bootstrap samplings are present. Also, the average of the 10,000 mean yield anomalies is referred as the averaged anomalies in the positive/negative event years, and its significance is based on the bootstrap probability or *p*-value. The *p*-value reflects the percentage of 10,000 samplings when the mean yield anomalies in the event years are larger/smaller than 0. For example, if the mean yield anomalies in the event years are larger/smaller than 0 for 90% cases of 10,000 samplings, the *p*-value is regarded as 0.1.

## Additional Information

**How to cite this article**: Yuan, C. and Yamagata, T. Impacts of IOD, ENSO and ENSO Modoki on the Australian Winter Wheat Yields in Recent Decades. *Sci. Rep.* **5**, 17252; doi: 10.1038/srep17252 (2015).

## Supplementary Material

Supplementary Information
