# Supplementary material for: Impacts of IOD, ENSO and ENSO Modoki on the Australian Winter Wheat Yields in Recent Decades
Source: Sci Rep. 2015 Nov 30;5:17252. doi: 10.1038/srep17252 (PMC4663488; doi:10.1038/srep17252)
Supplement: Supplementary Information [file srep17252-s1.pdf]

Supplementary Information

Impacts of IOD, ENSO and ENSO Modoki on the Australian Winter Wheat Yields in Recent Decades

Chaoxia Yuan[1,2] and Toshio Yamagata[2]

[1]*Key Laboratory of Meteorological Disaster of Ministry of Education, Collaborative Innovation Center on Forecast and Evaluation of Meteorological Disasters, Nanjing University of Information Science & Technology, Nanjing 210044, China*

[2]*Application Laboratory, JAMSTEC, Yokohama 236-0001, Japan*

The data used for the supplementary figures include the wheat yield data downloaded from the Australian Bureau of Statistics, the Global Precipitation Climatology Project monthly precipitation (GPCP)[28], and OISST from January 1982 to December 2011.

28. Adler, R. F. et al. The version 2 global precipitation climatology projection (GPCP) monthly precipitation analysis (1979-Present). *J. Hydrometeor*. **4**, 1147-1167 (2003)

Supplementary Table 1. The positive and negative years of IOD, canonical ENSO and ENSO Modoki based on the SON DMI, NDJ Niño3 and JJA EMI when they are over 0.7 standard deviation of DMI, Niño3 and EMI in the last three decades. We note that the present particular seasonal definition of climate mode years is different from conventional definition. The years when El Niño (La Niña) or El Niño (La Niña) Modoki co-occurred with the positive (negative) IOD are underlined. We note

	Positive	Negative
IOD	1982, 1991, 1994, 1997, 2002, 2006, 2007	1984, 1989, 1990, 1992, 1993, 1996, 1998, 2001, 2005, 2010
ENSO	1982, 1986, 1987, 1991, 1994, 1997, 2002, 2006, 2009	1983, 1984, 1985, 1988, 1995, 1996, 1998, 1999, 2000, 2005, 2007, 2010
ENSO Modoki	1982, 1991, 1992, 1994, 2002, 2004	1983, 1997, 1998, 1999, 2008, 2010

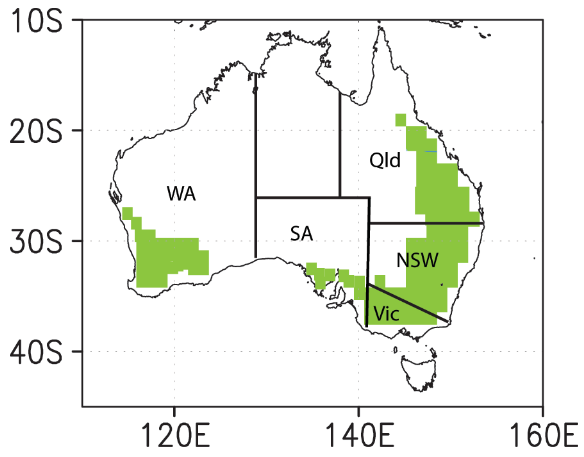

Supplementary Figure 1: Australian wheat belt (green shading) and five major wheat-producing provinces, Western Australia (WA), Southern Australia (SA), Victoria (Vic), New South Wales (NSW) and Queensland (Qld). The figure was drawn by Grads and Adobe Illustrator CS4.

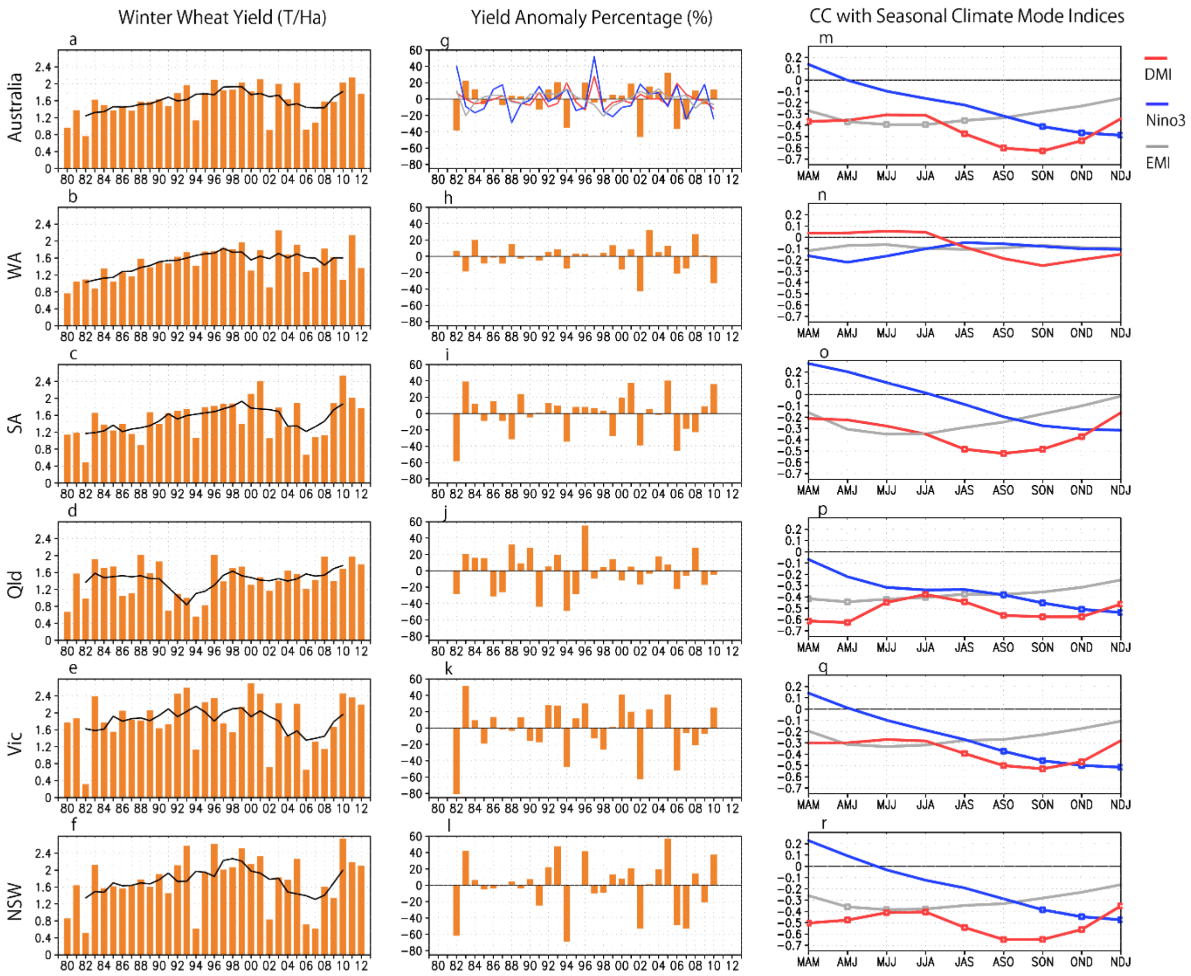

Supplementary Figure 2: Time series of (a-f) winter wheat yields, (g-l) their year-to-year anomalous percentages (%) and (m-r) correlation coefficients with the three-month-running mean indices of IOD (DMI, red line), ENSO (Niño3, blue line) and ENSO Modoki (EMI, gray line) in Australia and its five major wheat-producing provinces, WA, SA, Qld, Vic and NSW. The correlation coefficients significant at the 95% confidence level are marked by the open circles in m-r. The September-November DMI (red, ˚C), November-January Niño3 (blue, ˚C) and June-August EMI (gray, ˚C) are superimposed in g after multiplied by 15. Years in the X axis denote the years when the wheat is sowed. The figure was drawn by Grads.

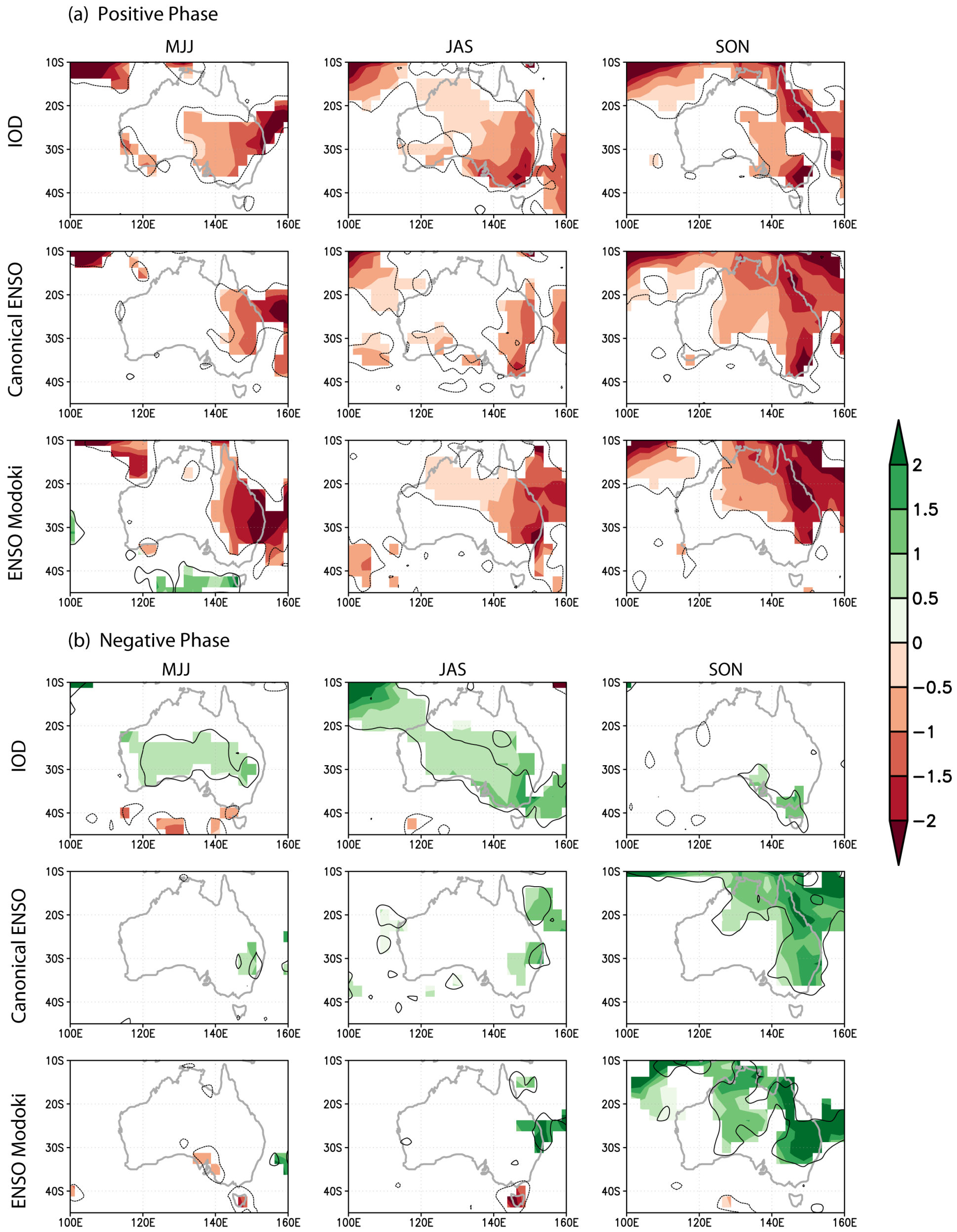

Supplementary Figure 3: Composite anomalies of precipitation (mm day^{-1}) in the positive (a) and negative (b) years of IOD, canonical ENSO and ENSO Modoki in May-July (MJJ), July-September (JAS) and September-November (SON). Shading (contour) denotes the anomalies significant at the 80% (90%) confidence level. The event years are consistent with Supplementary Table 1. GPCP precipitation data are used here. The figure was drawn by Grads.

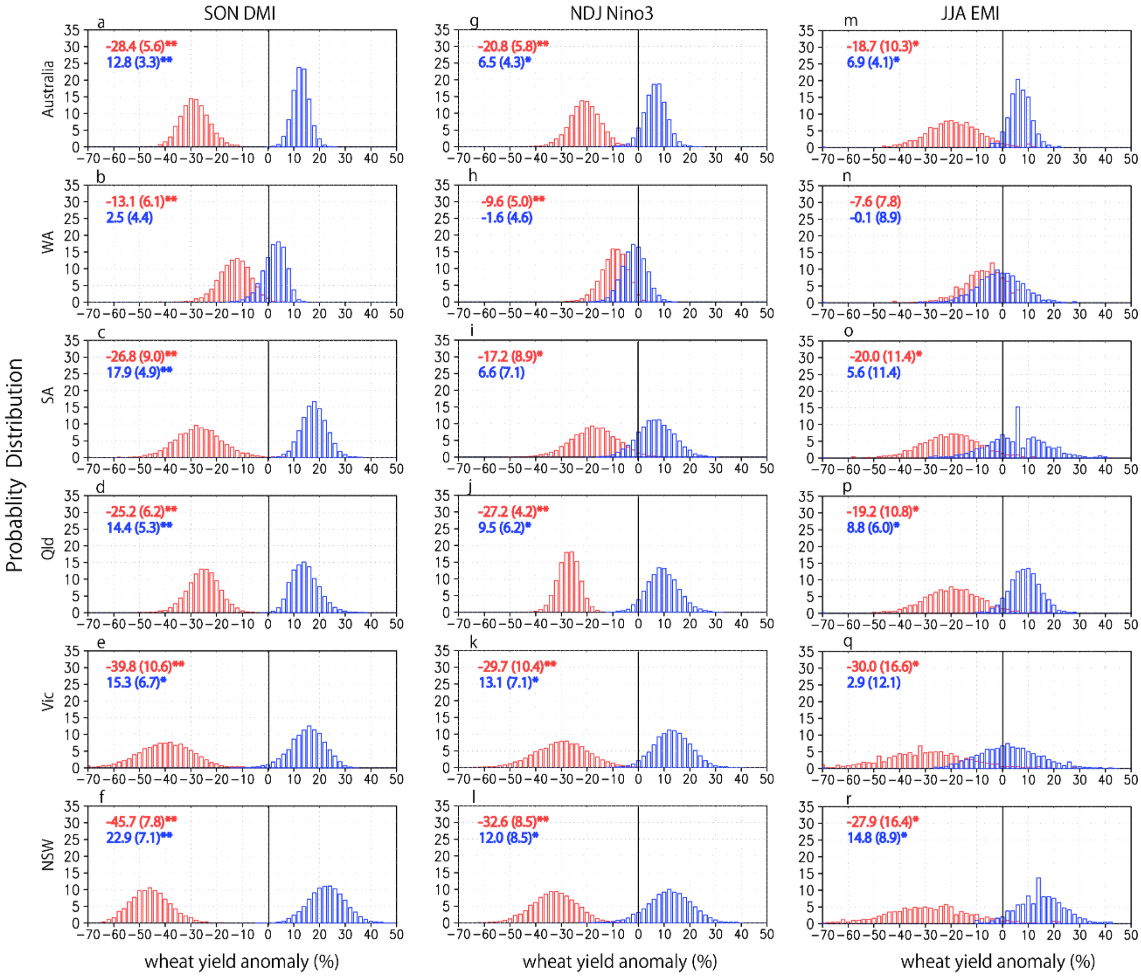

Supplementary Figure 4: Probability distribution (%) of the mean wheat yield anomaly (%) in Australia and its five major wheat-producing provinces in the positive (red) and negative (blue) years of (a-f) IOD, (g-i) canonical ENSO and (m-r) ENSO Modoki after 10,000 bootstrap samplings. The positive and negative years are selected based on the 0.7 standard deviation of September-November DMI, November-January Niño3 and June-August EMI, respectively. Red and blue numbers with (without) parenthesis in each panel denote the average (standard deviation) of mean yield anomalies in the positive and negative event years. Double (single) asterisks denote the confidence level at 99% (90%). The figure was drawn by Grads.

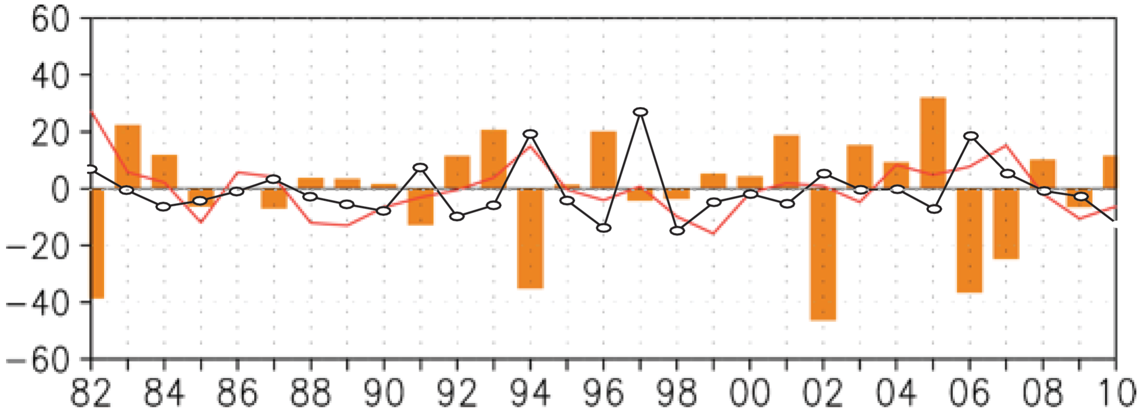

Supplementary Figure 5: Times series of (orange bar) the observed Australian winter wheat yield variations (%), (dark line) observed September-November IOD indices (˚C) and (red line) the predicted September-November IOD indices (˚C) by SINTEX-F with the initialization on April 1st. Both observed and predicted IOD indices have been multiplied by 15. The figure was drawn by Grads.

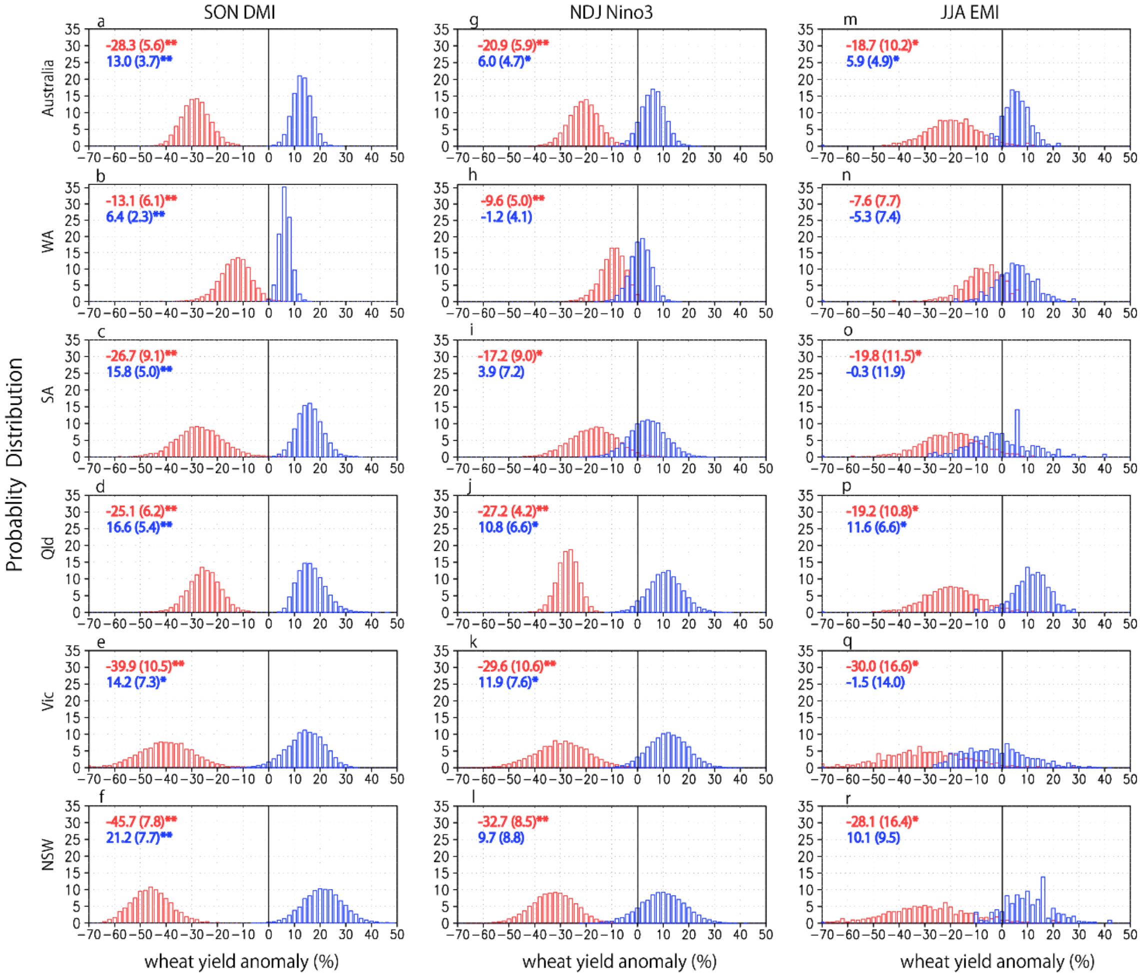

Supplementary Figure 6: As in Supplementary Fig. 4, but the year of 2010 is excluded. The figure was drawn by Grads.
